# Preliminary evidence for an increased likelihood of a stable trajectory in mild cognitive impairment in individuals with higher motivational abilities

**DOI:** 10.1186/s12877-018-0865-5

**Published:** 2018-08-13

**Authors:** Myriam V. Thoma, Simon Forstmeier, Roger Schmid, Oliver Kellner, Franziskos Xepapadakos, Ursula Schreiter Gasser, Andreas Blessing, Axel Ropohl, Gabriela Bieri-Brüning, Dries Debeer, Andreas Maercker

**Affiliations:** 1 University of Zurich, Binzmuehlestr, 14, 8050 Zurich, Switzerland; 2 University Research Priority Program “Dynamics of Healthy Aging”, University of Zurich, Zurich, Switzerland; 3 University of Siegen, Faculty II, Developmental Psychology, Adolf-Reichwein-Str. 2, 57068 Siegen, Germany; 4 Psychiatrische Klinik Zugersee, Zug, Switzerland; 5 Integrierte Psychiatrie Winterthur, Winterthur, Switzerland; 6 Present Address: Alterspsychiatrische Praxis, Buehlach, Switzerland; 7 Clienia Schloessli AG, Oetwil am See, Switzerland; 8 Praxis für Psychiatrie Rehalp, Zurich, Switzerland; 9 Psychiatrische Dienste Thurgau, Muensterlingen, Switzerland; 10 Sanatorium Kilchberg AG, Kilchberg, Switzerland; 11 Present Address: KMG – Kompetenz mentale Gesundheit GmbH, Baar, Switzerland; 12 Geriatric service of the city of Zurich, Zurich, Switzerland; 13 Psychopathology and Clinical Intervention, University of Zurich, Binzmuehlestr. 14/17, CH-8050 Zurich, Switzerland

**Keywords:** Mild cognitive impairment, Stability, Informant-rated motivational abilities

## Abstract

**Background:**

Motivational abilities (MA), that describe skills in relation to goal-oriented behavior, have recently been found to be associated with neuropathological aging. Here we examine the impact of MA on the long-term course of mild cognitive impairment (MCI).

**Methods:**

We followed-up *N* = 64 individuals diagnosed with MCI (*M*_age_ = 73 years, 44% female) for 3 years. MA were assessed by long-term informants of the participants using two scales: motivation and decision regulation [Volitional Components Questionnaires, VCQ, (Kuhl and Fuhrmann, Decomposing self-regulation and self-control: the volitional components inventory, 1998)]. Cognitive abilities were assessed with the Mini Mental State Examination (J Psychiatr Res 12:189-98, 1975). Survival analyses and multilevel modeling (MLM) were applied to determine the predicting effect of informant-rated MA at baseline on the likelihood of MCI stability and on the trajectory of cognitive abilities.

**Results:**

Fifty percent (*n* = 32) of the MCI participants remained stable, while 32.8% (*n* = 21) and 17.2% *(n* = 11) converted to Alzheimer’s disease (AD) or dropped-out, respectively. Survival analyses revealed that MCI cases with higher-rated MA at baseline were more likely to exert a stable course in MCI over 3 years (*p* = 0.036) when controlling for demographic characteristics and executive function. MLM analyses indicated that higher informant-rated MA at baseline were significantly related to higher cognitive abilities, even when controlling for MCI subtype (*p* = 0.030).

**Conclusions:**

This study provides preliminary longitudinal evidence for a lower risk of conversion to AD and higher cognitive abilities by higher rated MA at an early stage of MCI.

## Background

Mild cognitive impairment (MCI) is defined as an objectified decline of cognitive abilities in an individual over time in at least one cognitive domain, while independence with regard to functional abilities prevails and dementia criteria are not met [1]. Alzheimer’s disease (AD) dementia is a slowly progressive neurodegenerative disorder including a gradual cognitive decline in more than one cognitive domain, most common is the amnestic syndrome, and in functional abilities of daily living [2]. AD differs from other dementia by the ‘cause’ of the dementia, i.e., the underlying pathophysiological changes within the brain. On a biochemical level, the accumulation of the amyloid-beta peptide (A*B*) in the brain has been identified as a crucial element in the pathology of AD [3].

While in many AD cases, MCI can be identified as a prodromal syndrome, not all MCI cases progress to AD or other types of dementia [4]; rather, some cases remain stable or even revert to previous cognitive performances (e.g., [5]). However, an increased understanding of why individuals differ in their stability or progression of MCI, i.e., what factors predict favorable courses, rather than a further decrease in cognitive abilities and thus conversion to dementia, would provide crucial indications about how preventive measures and therapeutic interventions could best be applied.

This study has been conducted to understand the predictive role of motivational abilities on the course of MCI. Given the strong link to current brain and cognitive reserve models, we start with describing these models in more detail. In a next step, we provide an extensive definition of motivational abilities, as those build the basis for the motivational reserve (MR) model. Due to its relatively new emergence into the scientific literature, we further describe the MR model and the rationale behind its supposedly protective effect against cognitive decline. For this, we provide an overview over previous studies conducted with cognitively intact and cognitively impaired older adults. Finally, we present our main research question.

### Brain and cognitive reserve and resilience

Dementia progression is characterized by a substantial interindividual heterogeneity that cannot be sufficiently explained with disease characteristics [6]. Contextual factors, including social aspects, psychological and physical factors have to be taken into account when aiming to predict progression of dementia [6]. Previous research indicates that living a physically, socially and intellectually stimulating and active life may have a protective impact on the pathophysiological process of MCI and AD (for an overview see [7]). This phenomenon became known as the so-called brain or cognitive reserve [8–10]. The cognitive reserve model builds upon the epidemiological observation that despite the presence of AD neuropathology, some older individuals do not show clinical signs of cognitive impairment (e.g. [11]). Within a new framework proposed by Arenaza-Urquijo and Vemuri [12] this phenomenon that has been described as ‘resilience‘to AD, i.e., normal cognitive functioning despite underlying substantial AD pathologies (versus ‘resistance‘i.e., normal cognitive functioning without/low underlying substantial AD pathologies). According to the cognitive reserve model, the available reserve within the brain (of hereditary or acquired origin, or both) can tolerate or absorb neurodegenerative processes before the underlying pathophysiological process becomes clinically manifested [13]. The higher the reserve within the brain, the more neuropathology is required in order to interfere with cognition and functional abilities [14].

While the brain and cognitive reserve models are crucial in the understanding of interindividual differences in the susceptibility for developing clinically relevant AD pathology [14], the current models lack to acknowlegde factors that influence the “whys” an individual initiates and carry out those activites in the first place. This question relates to motivational abilities, that lie at the bottom of such behavior [15].

### Motivational abilities

Motivational abilities are crucial for the understanding of the trajectory of an individual’s personal and professional life as they are skills necessary to pursue goals. The way an individual pursues a particular goal, changes as a function of age. Several motivational theories exist that focus on variabilities in goal management over the life-course, such as the dual-process framework [16], the model of selection, optimization and compensation [17] or the life-span theory of control [18].

In general, two different stages can be differentiated in the pursuit of goals: the stage of selecting a particular goal (=goal-setting phase) and the stage of goal-striving [19]. For the goal-setting phase, aspects of control and expectancy are needed. A prominent concept related to this is the self-efficacy concept by Bandura [20] which describes the strength of belief one has in his or her own capabilities to deal with life’s challenges. For the goal-striving phase, aspects of volition and self-regulation are of importance, such as motivation, decision and activation regulation. Motivation regulation refers to the ability to keep oneself motivated when difficulties arise [21]. Decision regulation describes the ability to make self-congruent decisions [21]. Activation regulation finally is essential to initiate activities planned to reach a set goal [22]. These regulation processes are illustrated by the following hypothetical example: decision regulation processes are required when an individual makes the decision to register for an exam which is mandatory to graduate from college (= self-congruent goal). The repeated process to activate oneself to study for this exam, rather than spending time with friends, requires activation regulation processes. To keep up with the study plan despite adverse circumstances, such as also having to work night shifts, requires motivation regulation. Finally, the belief that one can achieve the set goal despite a potentially unfavorable environment involves self-efficacy. Together, motivational abilities are involved in the phases of setting and striving a particular goal and are of thus integral part of goal-directed human behavior.

### Possible mechanisms underlying the association between motivational reserve and cognitive trajectories

It follows that motivational aspects are intrinsically connected to activities and behaviors traditionally associated with cognitive reserve. This notion is supported by previous empirical work in varying areas. Objective career success has been found to be meaningfully related to motivation [23]. Self-motivation, expected benefits, intention to exercise are all relevant aspects in explaining “why” an individual engages in physical activity [24]. It becomes obvious that motivation (‘will’) and cognition (‘skill’) are both crucial factors for achievements by self-regulated learning [25]. Given this close link between the psychological entities of motivation and cognition [26] “cognitive” activities or behaviors may not be examined in isolation of motivational abilities that lie at the bottom of such behavior. Given the intrinsic connection between motivation and cognition, our research group expanded existing reserve models with motivation-related aspects (motivational reserve [MR], [27]).

The basic assumption of the MR model is that motivational abilities of an individual exert a protective influence against neuropathological damage. The assumed underlying mechanism is believed to work in analogy to the brain or cognitive reserve concepts [8–10, 28]. With regard to possible mechanisms, current understanding is that an adequate stimulation of the brain through particular experiences, training or exercise of certain abilities, such as within the context of education, occupation or leisure activities throughout the lifespan is a prerequisite to increase cognitive reserve [14]. The suggested underlying neural basis discussed hereby are the enhancement of the interconnectivity of various brain networks in form of higher efficiency and capacity and compensatory mechanisms [14, 29]. The increased use of brain areas specifically related to motivational abilities is assumed to promote synapse formation and neuronal growth, which then lead to higher efficiency within the brain, that in turn allows the compensation of impaired neuronal networks [30, 31]. For instance, self-efficacy has been found to predict performances in a memory task after 6 years in a sample of older adults [32]. Or internal locus of control, which describes the confidence that one’s action lead to a particular outcome, a construct with strong similarities to self-efficacy [33], were found to be related to the volume of the hippocampus in older adults [34]. A potentially indirect mechanism may also be assumed by which motivation influences the experience of stress, and promotes emotional health or cognition [30, 31].

### Previous research on the association between midlife motivational abilities and cognitive decline

Previous research efforts provided first evidence of a meaningful association between higher motivational abilities and decreased cognitive decline. In a cross-sectional study with non-demented individuals of 60-years of age and older, it was examined whether midlife motivational abilities, that were estimated on the basis of participant’s professional history, were related to cognitive status, well-being and the risk to develop MCI [30]. For this, the *Occupational Information Network* (O*Net; [35, 36]) was used to code all occupations a participant had in his / her professional life. For each profession, motivational abilities necessary to conduct the job were rated by two individual raters (for an extensive description of the exact method, see [30]). It was found that motivation-related occupational abilities were related to the risk of MCI, as well as cognitive status and well-being [30]. This was found even when factors were controlled that are generally related to cognitive reserve concepts, such as education and cognitive ability. In a next step, our research group took this question one step further and examined the same research question in the context of a prospective study with a larger (over 2000 older adults) and older (age of 75-years or older), non-demented sample [31]. It was found that high motivation-related occupational abilities were related to reduced risk (− 35%) to develop MCI and AD (in ApoE epsilon4 carriers only, [31]). These studies provided initial support that motivational abilities are associated in a meaningful way with the risk for the development of cognitive decline.

### Predictive effect of current motivational abilities on cognitive decline

It remains unclear whether motivational abilities can be used to predict the course of existing cognitive decline. It is assumed that individuals with higher motivational abilities may exert a more stable course as compared to individuals with lower motivational abilities due to their higher reserve.

Palmer and colleagues showed that motivational abilities may still exert a protective influence in the context of existing cognitive decline [37]. This study was centered around the syndrome apathy, which includes a lack of motivation in its set of criteria [38]. The authors reported significant increases in the risk for conversion from MCI to AD in apathetic patients [37].

### Goals of the present study

In previous studies, a retrospective estimate of motivational abilities has been used to examine the potentially protective effect of motivational abilities on cognitive decline [30]. This retrospective estimate was created by rating midlife motivational abilities on the basis of a participant’s occupational history (O*Net; [35, 36]). While this method showed to be a valuable approach to estimate midlife motivational abilities, they are nonetheless indirect measures. In the presence of a lack of assessment of motivational abilities in midlife, which would have been the optimal measure for the use as predictor, what are acceptable alternatives? Retrospective assessments suffer from recall biases (e.g. [39]). What is more, given the reduced abilities of accurate self-judgements in individuals with cognitive decline [40] current self-reports of one’s abilities may be less accurate. We therefore chose informant-rated motivational abilities as predictors for this study to examine whether they can be used to predict the long-term course of MCI.

Therefore, we aimed to examine the hypothetically protective influence of current motivational abilities that were rated by informants of the participants, on the stability of MCI. In this study, we focused on the motivational abilities ‘decision regulation’ (= to decide to do a specific task) and ‘motivation regulation’ (= to stay with the task). These two motivational abilities were chosen as, in contrast to the rather robust self-efficacy ‘belief’, they are modifiable and thus well applicable for potentially future intervention. From a content-related as well as methodological perspective, decision and motivation regulation belong together and should as such be assessed in combination, without the additional assessment of other motivation-related constructs (e.g. activation regulation). We hypothesized that individuals with higher-rated motivational abilities would exhibit an increased likelihood for stability of MCI, as compared to MCI individuals with lower informant-rated motivational abilities, when followed-up for a period of 3 years.

## Methods

The data presented here have been sampled in the context of the longitudinal study “Motivational Reserve as a Protective Factor in Mild Alzheimer’s Dementia and Mild Cognitive Impairment” (MoReA, [41]).

### Study participants

For recruitment and diagnostic purposes, we cooperated with several different German-speaking Swiss and Austrian memory clinics and specialized institutions for cognitive impairment and dementia. Diagnostic assessments were conducted by neuropsychologists and medical doctors at the respective institutions, independently, and before the partaking of the participants in the current study. In each of the cooperating memory clinics, diagnoses for participants were made in consensus in an interdisciplinary team, after an extensive psychiatric, neurological, clinical and neuropsychological assessment (see detailed diagnostic assessment below). Inclusion criteria for study participation were as follows: willingness to partake in the longitudinal study, minimum age of 60 years, and the clinical diagnoses of MCI and mild AD. For this study, only individuals diagnosed with MCI were included. Exclusion criteria applied in the MoReA-study were the diagnosis of a pure vascular dementia or diagnoses of Parkinson’s, Creutzfeld-Jakob’s or Pick’s disease, as well as traumatic injuries to or previous surgeries of the brain, as well as neurological diseases or syndromes such as epilepsy, post-encephalitic or post-concussional syndromes. Further exclusion criteria were chronic diseases (such as HIV, metabolic or hematologic disorders), severe organ failure, history of malignant diseases or current psychiatric disorders other than mild AD. Potential participants and their informal caregivers were informed about the study by the clinical experts at the specialized clinics and institutions. In case of expressed interest by the patients to partake in the study, participants had to complete a written informed consent at the respective clinic allowing the project researchers to contact them. Contact details, diagnostic information and neuropsychological data were provided to the researchers. Together, the memory clinics referred *n* = 133 individuals to the study center (= baseline participation rate). Almost 30% (*n* = 22) refused at that stage to participate in the study. Reasons for non-participation were excessive demands after learning about the diagnosis, or the informants had no time to take part in the study.

### Informants

In order to be able to participate in the study, patients were required to be accompanied by an informant, i.e., a reliable source of information. Those informants were predominantly partners of the participants (70%), children (17%), or other relatives and friends of the patients (13%). Most of the informants see the patients on a daily or almost daily basis (73.6%) or several times per week (5.7%). On average, informants know the patients since 46 years (SD = 14.02), with a range of 6 to 70 years. Written informed consent was obtained from all informants.

### Procedure

#### Baseline assessment (time 1, t1)

A first appointment was arranged with the patients and their informants, during which in-depth information with regard to content and procedure of the study was provided by the researchers. In the case of persisting interest, the patients and their informants both provided written informed consent. During the remaining time of this first appointment, data collection started, separately for the patient and the informant, in two different rooms. Neuropsychological, psychiatric, motivational, cognitive and socio-demographic information were assessed during the second part of the first meeting and completed on a second meeting, an approximately 2.5 h appointment conducted within the next week.

#### Follow-ups (t2, t3, t4)

Clinical, neuropsychological and cognitive data were re-assessed during meetings of approximately 1.5–2 h, separated by 12-month intervals. Data sampling was executed in a similar fashion as on the baseline assessment (i.e., separately for the patient and the informal caregiver). The informal caregiver was interviewed once per follow-up. All patient interviews over the course of the 3 years were conducted by the same interviewer. This research assistant has also been the constant contact person for the patients and informal caregivers. The interviewer for the informal caregivers varied. For the assessment at t1, as well as for all follow-ups, participants were reimbursed with 50 Swiss Francs. The ethics committees of the Zurich cantonal medical authority approved the research protocol.

### Diagnostic assessment of MCI and AD

An extensive battery of diagnostic instruments for MCI and AD has been used in the MoReA study (for a complete overview see, [41]). In the following, we list those instruments that have been applied in the present analyses.

Taking into account the international consensus criteria [42], MCI was operationalized in the current study as following: a) not meeting the DSM-IV criteria for dementia (Mini Mental State Examination, MMSE, score of 24 points or higher, [43]), b) cognitive decline reported by the patient and / or informant, and / or quantifiable deteriorating cognitive abilities over time, c) no impairment in basic activities of daily living and only minimal impairment in complex instrumental functions (Clinical Dementia Rating Scale, CDR, required score of 0.5 points, [44]) and d) impairment (at least mildly) in one or more of the subsequent domains: memory, executive function, attention, language and praxis. The criterion for cognitive decline was > 1.5 standard deviation impairment compared to age and education corrected norm scores.

The diagnostic criteria given by the *Diagnostic and Statistical Manual of Mental Disorders* (DSM-IV-TR, American Psychiatric Association, [45]) and those of the *National Institute of Neurological and Communicative Disorders and Stroke-Alzheimer’s Disease and Related Disorders Association* (NINCDS-ADRDA, [2]) were used for the diagnosis of probable AD. The NINCDS-ADRDA criteria require a history of cognitive decline and evidence of impairment in memory and at least one other cognitive domain. Possible AD cases (in NINCDS-ADRDA terminology) were also included, i.e., persons who met these criteria and also had another condition thought to be contributing to cognitive impairment. Diagnostic criteria for AD involved an insidious onset and observable progression or quantifiable worsening of cognitive impairment (in memory and at least in one other cognitive domain) and meaningful functional impairment. Mild AD cases were selected on the basis of a CDR score of 1 [46] and MMSE score between 18 and 28 points (DSM-IV-TR, American Psychiatric Association, [45]).

For the assessment of cognitive function, we applied the *Consortium to Establish a Registry for Alzheimer’s Disease – Neuropsychological Assessment* NINCDS-ADRDA, (CERAD-NP, [44]). The CERAD-NP consists of the MMSE [43] and an additional 13 measures related to cognitive functioning (for a complete overview see, [41]). To diagnose and determine the severity of AD, we applied the widely used, semi-structured CDR interview [44].

### Classification of participants into subgroups

The participants that were diagnosed with MCI at both t1 and all follow-ups until t4 (i.e., those participants that remained stable with regard to their cognitive decline throughout the observation period of 3 years), were classified into the subgroup ‘*MCI stability’.* Those participants that converted to (any degree of severity of) AD by the assessment at t4, were classified into the group *‘MCI conversion’.*

### Psychometric assessment of motivational abilities – main predictor

We further assessed a composite measure of patients’ motivational abilities at baseline (= current motivational abilities) through informant-ratings of two different scales for motivation and decision regulation. Two scales of the *Volitional Components Questionnaires* (VCQ) [21] were applied to measure ‘motivation regulation’ (e.g., “I can usually motivate myself quite well if my determination to persevere weakens”; Cronbach’s alpha = .87) and ‘decision regulation’ (e.g., “When I think about doing or not doing something, I usually arrive at a decision quickly”; Cronbach’s alpha = .69).

The original scales are all constructed as self-report measures. To receive informant-rated measures, we reformulated the original items in such a way that the item can be applied as informant-rated measures (e.g., original item “I can usually handle whatever comes my way”; re-formulated item “He/she can usually handle whatever comes his / her way”). There is a small to moderate correlation between informant and self-rated motivational abilities [41].

To summarize, a composite measure including two scales, which were z-transformed beforehand, was created for the assessment of the current informant-rated motivational abilities of the participants.

### Additional variables

General information, including living situation, family status, education, former occupation and health, were assessed with self-reports. For an extensive description of former occupation of the sample and how it was assessed, we refer to Forstmeier and Maercker [41]. Rather than a measure for physical health in general, we assessed risk factors with regard to cardiovascular health, as those have previously been found to meaningfully increase risk for AD (e.g. [47]). We assessed following factors to calculate a proxy for cardiovascular risk: diagnosis of diabetes mellitus, diagnosis of elevated cholesterol and high blood pressure. Out of these variables we calculated a composite measure for cardiovascular risk.

To control for depression, we applied the German version of the *Geriatric Depression Scale* (GDS, [48, 49]). The Cronbach’s alpha of 0.91 indicates a high internal consistency [48]. As protective factor, we included education (numbers of years of education) as a proxy for cognitive reserve. To control for the effect of higher or lower executive functions on MCI stability or conversion or the trajectory of cognitive abilities, we included the Stroop-Color Word test [50].

### Statistical analyses

All statistical analyses were performed using SPSS for Mac OSX (22.0) software packages (IBM, Chicago, IL, USA). Descriptive analyses were used for the calculation of mean (M) and standard deviation (SD). To assess potential demographic differences between groups, t-tests, and chi-square tests were applied. Bivariate correlations were performed to identify relationships between variables of interest. Little’s missing completely at random (MCAR) test was conducted to test whether data was missing in a random way [51]. All data was found to be MCAR. Multiple imputation was used to address missing data. The incomplete variables were imputed variable by variable: a filled-in variable is then used as a predictor in subsequent steps. SPSS automatically used the monotone method (multiple imputation algorithms). SPSS applies sequential regression imputation, i.e., linear and logistic regression for continuous variables and categorical variables, respectively. Diagnoses of drop-outs were not imputed. The variables age, sex, MCI subtype, education (years) had no missing values. The variables ‘motivational abilities’, ‘children’, and ‘cardiovascular risk factors’ had 1.89, 15.09, and 24.53% missing values, respectively. Analyses of whether conclusions depend on the imputed variable revealed only minimal differences between the imputed and non-imputed data set with no change in the conclusions.

The analytic method of survival analysis using logistic regression has been chosen to predict which patients are most likely to stay stable, i.e., keep MCI diagnosis within the observation period of 3 years [52]. Hosmer and Lemeshow test results were used to test whether the models were a good fit for the data. An odds ratio (OR) lower than 1 is indicative of an increased risk for conversion, while an OR higher than 1 indicates a reduced risk for conversion. Given the limited power due to the relative low number of observations and the data reduction due to dichotomizing into ‘MCI stability’ and ‘MCI conversion’, we added additional calculations with multilevel modeling [53]. Multilevel modeling was used to test whether the trajectory in cognitive abilities, as measured with the MMSE, can be predicted by informant-rated motivational abilities. Our sample size for multilevel modeling is sufficient for an accurate estimation [54, 55]. Multilevel modeling was conducted with a non-imputed data set. As a measure of fit, the Akaike information criterion (AIC) was applied. Smaller values in the AIC are indicative of a better fit of a model [53]. For the calculation of R^2^ for multilevel modeling, we used calculations provided by Xu [56]. Data sets for the survival analyses as well as for the multilevel model were vertically arranged, resulting in number of observations rather than number of patients.

All reported results were considered to be significant at the *p* ≤ 0.05 level and were considered a trend at the *p* ≤ 0.1 level.

## Results

### Sample characteristics

At t1, the study included *n* = 64 MCI participants. Sample characteristics at t1 are shown without the inclusion of participants that dropped-out from the study (*n* = 11) within the observation period of 3 years (for sample characteristics at t1, see Table 1). Drop-out was not related to sample characteristics (e.g. age, sex), cardiovascular risk, cognitive and motivational abilities or depression at t1. Reasons for drop-out from t1 to t2 (*n* = 7) were: lost interest (*n* = 4), death (*n* = 2), and severe somatic illness (*n* = 1). Reason for drop-out from t2 to t3 was death (*n* = 1). Reasons for drop-out from t3 to t4 were: severe somatic illness (*n* = 1), excessive demand of the testing situation (*n* = 1) and not reachable (*n* = 1).

**Table 1:** Sample characteristics at baseline of Mild Cognitive Impairment (MCI) cases, excluding drop-outs (*n* = 53), and comparison of basic characteristics between subgroups ‘MCI Stability’ (*n* = 32) and ‘MCI Conversion’ (*n* = 21) at last follow-up (t4)

Variables	MCI total *(n* = 53)	MCI stability (*n* = 32)	MCI conversion (*n* = 21)	*t* / *χ*^*2*^*-*test	*p*	*d / phi*
Age (years), *M* (SD)	73.09 (7.12)	71.16 (7.61)	76.05 (5.19)	2.57 (*t*)	**0.013**	−0.75 (*d*)
Sex, female, *n* (%)	26 (49.1)	20 (62.5)	6 (28.6)	5.84 (*χ*^*2*^)	**0.016**	−0.33 (phi)
Education (years), *M* (SD)	12.32 (2.52)	12.09 (2.37)	12.67 (2.75)	−0.95 (*t*)	0.346	−0.23 (*d*)
Living Situation, *n* (%)				1.79 (*χ*^*2*^)	0.409	0.18 (phi)
- Alone	14 (26.4)	10 (31.3)	4 (19.0)			
- Partner	38 (71.7)	21 (65.6)	17 (81.0)			
- Family member	1 (1.9)	1 (3.1)	0			
Marital status, *n* (%)				1.54 (*χ*^*2*^)	0.674	0.17 (phi)
- Single	3 (5.7)	2 (6.3)	1 (4.8)			
- Married	38 (71.7)	21 (65.6)	17 (81.0)			
- Divorced /separated	4 (7.5)	3 (9.4)	1 (4.8)			
- Widowed	8 (15.1)	6 (18.8)	2 (9.5)			
Children, *M* (SD)	2.36 (0.88)	2.00 (0.68)	2.89 (0.90)	3.77 (*t*)	**< 0.001**	−1.12 (*d*)
MMSE score, *M* (SD)	27.25 (2.04)	27.69 (2.22)	26.57 (1.54)	−1.79 (*t*)	*0.081*	0.57 (*d*)
CDR, *M* (SD)	0.42 (0.19)	0.39 (0.21)	0.45 (0.15)	−1.25 (*t*)	0.218	−0.33 (*d*)
GDS-S, *M* (SD)	2.93 (2.3)	3.34 (2.55)	2.30 (1.97)	1.58 (*t*)	0.121	0.46 (*d*)
MCI Subtype, *n* (%)				11.76 (*χ*^*2*^)	**0.008**	0.47 (phi)
- Amnestic s-d	11 (20.8)	5 (15.6)	6 (28.6)			
- Amnestic, m-d	17 (32.1)	6 (18.8)	11 (52.4)			
- Non-amnestic, s-d	22 (41.5)	19 (59.4)	3 (14.3)			
- Non-amnestic, m-d	3 (5.7)	2 (6.3)	1 (4.8)			
Cardiovascular risk, *n* (%)						
- Diagn. diabetes mellitus	11 (20.8)	9 (28.1)	2 (9.5)	3.64 (*χ*^*2*^)	0.162	0.28 (phi)
- Diagn. elevated cholesterol	15 (28.3)	12 (37.5)	3 (9.4)	2.63 (*χ*^*2*^)	0.105	−0.25 (phi)
- Diagn. high blood pressure	22 (41.5)	16 (50)	6 (28.6)	1.21 (*χ*^*2*^)	0.545	0.16 (phi)

*CDR* clinical dementia rating scale, *d* Cohen’s d, *Diagn.* diagnosis, *GDS-S* geriatric depression scale – self-report, *M* mean, *MCI* mild cognitive impairment, *m-d* multi-domain, *MMSE* Mini-Mental State Examination, *n* number, *p* probability, *phi* Cohen’s phi, *SD* standard deviation, *s-d* single-domain, *t* independent t-test, *χ*^*2*^ chi-square test

The bold values indicate a statistically significant difference with a *p*-value less than 0.05

Clinical phenotypes of the *n* = 53 MCI patients at t1 were as follows: amnestic, single-domain (*n* = 11), amnestic, multi-domain (*n* = 17), non-amnestic, single-domain (*n* = 22), and non-amnestic, multi-domain (*n* = 3). From t1 to t4, 50% (*n* = 32) of the MCI participants remained stable (i.e., they kept their cognitive level), while 32.8% (*n* = 21) and 17.2% (*n* = 11) converted to AD or dropped-out, respectively (see Table 2). None of the MCI participants converted back to normal cognitive functioning within the observation period from t1 to t4.

**Table 2:** Number of Mild Cognitive Impairment (MCI) and Alzheimer Disease (AD) cases and drop-outs at baseline and follow-ups in number (*n*) and percent (*%*) (*n* = 64)

	*MCI*	*Mild AD*	*Moderate AD*	*Severe AD*	*Drop-out*
Baseline (t1)	First Follow-up (t2)				
*MCI (n = 64)*	*44 (68.8%)*	13 (20.3%)	0 (0%)	0 (0%)	7 (10.9%)
First Follow-up (t2)	Second Follow-up (t3)				
*MCI (n = 44)*	38 (86.4%)	6 (13.6%)	0 (0%)	0 (0%)	0 (0%)
*Mild AD (n = 13)*	0 (%)	10 (76.9%)	2 (15.4%)	0 (0%)	1 (7.7%)
Second Follow-up (t3)	Third Follow-up (t4)				
*MCI (n = 38)*	32 (84.2%)	4 (10.5%)	1 (2.6%)	0 (%)	1 (2.6%)
*Mild AD (n = 16)*	0 (%)	11 (68.6%)	2 (12.5%)	1 (6.3%)	2 (12.5%)
*Moderate AD (n = 2)*	0 (%)	0 (%)	0 (%)	2 (100%)	0 (%)
Baseline (t1)	Third Follow-up (t4)				
*MCI (n = 64)*	32 (50%)	15 (23.4%)	3 (4.7%)	3 (4.7%)	11 (17.2%)

*AD* Alzheimer’s disease, *MCI* mild cognitive impairment, *n* number, *t1* baseline, *t2* first follow-up, *t3* second follow-up, *t4* third follow-up

Table 3 is a correlation matrix of variables used for the main analyses (see Table 3). It shows that higher age was significantly related to a higher number of children, to lower executive function and MCI amnestic subtype. Female sex was related to higher motivational abilities and lower education. Higher motivational abilities were related to fewer years of education (see Table 3).

**Table 3:** Correlation matrix of all predictors included in survival analyses using binary logistic regression and multilevel model (*n* = 64)

	(1)	(2)	(3)	(4)	(5)	(6)	(7)
(1) Age							
(2) Sex (0 = male, 1 = female)	−0.20 (0.11)						
(3) Number of children	**0.29 (0.03)**	− 0.10 (0.47)					
(4) Motivational abilities ^a^	− 0.09 (0.48)	**0.25 (0.05)**	0.04 (0.79)				
(5) Executive function ^b^	**0.28 (0.03)**	−0.07 (0.58)	0.14 (0.31)	−0.004 (0.97)			
(6) Education (years)	0.02 (0.88)	**−0.33 (0.01)**	0.22 (0.11)	**−0.25 (0.05)**	− 0.23 (0.07) ^d^		
(7) MCI subtype (0 = a; 1 = n-a.)	**− 0.30 (0.02)**	0.21 (0.10)	− 0.18 (0.18)	− 0.22 (0.09) ^d^	− 0.22 (0.09) ^d^	0.10 (0.46)	
(8) Cardiovascular risk ^c^	0.24 (0.10)	0.05 (0.76)	−0.08 (0.64)	0.11 (0.48)	0.10 (0.50)	0.03 (0.84)	0.17 (0.27)

The bold values indicate a statistically significant correlation with a *p*-value less than 0.05

*a* amnestic, *MCI* mild cognitive impairment, *n-a.* non-amnestic

^a^ Composite measure including informant-rated motivational abilities at baseline (t1)

^b^ Interference score of the Stroop Color-Word Test

^c^ Composite measure including the diagnosis of diabetes mellitus, elevated cholesterol and high blood pressure

^d^ marginally significant

Values in brackets indicate *p*-values

### Comparison of the subgroups of ‘MCI stability’ and ‘MCI conversion’

In a next step, we compared the basic characteristics of the ‘MCI stability’ and ‘MCI conversion’ subgroups. We identified significant differences between these two subgroups with regard to age, sex, MCI subtype and number of children (Table 1). The participants in the ‘MCI stability’ subgroup were generally younger, of female sex, of non-amnestic MCI subtype and having fewer children. With regard to informant-rated motivational abilities, groups differed significantly with regard to the composite measure, as well as with regard to motivation and marginally with regard to decision regulation (Table 4). Over all these measures, informants rated the current motivational abilities of the participants in the ‘MCI stability’ group higher as compared to informants of the participants in the ‘MCI conversion’ group.

**Table 4:** Informant-rated motivational abilities for all Mild Cognitive Impairment (MCI) cases at baseline (*n* = 53) and comparison of informant-rated motivational abilities between the subgroups ‘MCI Stability’ and ‘MCI Conversion’ as diagnosed at last follow-up (t4)

Motivational abilities ^a^	MCI total (*n* = 53)	MCI stability (*n* = 32)	MCI conversion (*n* = 21)	*t*-test	*p*	*d*
Composite measure, min-max, *M* (SD)	2–14.5	3–14.5	2–11	2.31	**0.025**	0.665
	8.48 (2.88)	9.21 (2.93)	7.40 (2.50)			
Motivation regulation, min-max, *M* (SD)	2–15	2–15	2–11	2.23	**0.030**	0.654
	8.37 (3.24)	9.16 (3.53)	7.19 (2.38)			
Decision regulation, min-max, *M* (SD)	0–15	0–15	2–12	1.92	*0.061* ^b^	0.545
	8.60 (3.10)	9.26 (3.10)	7.62 (2.91)			

*d* Cohen’s *d*, *M* mean, *max* maximum, *min* minimum, *n* number, *SD* standard deviation, *t* independent t-test

^a^ Composite measure including informant-rated motivational abilities at baseline (t1)

^b^ marginally significant

The bold values indicate a statistically significant difference with a *p*-value less than 0.05

### Prediction of MCI stability applying survival analyses using binary logistic regression

Survival analyses have been conducted using logistic regression to determine the predictive effect of informant-rated motivational abilities (= composite measure) at baseline (t1) on the likelihood of MCI stability (i.e., whether MCI participants remained stable with regard to their cognitive level over the course of 3 years). The differential predictive ability of the single components of the composite measure, i.e., motivation and decision regulation were also compared. Drop-outs were included in these analyses, resulting in *n* = 138 observations. Observations refer here to the information at each of the three follow-ups, of whether MCI patient remained stable or whether the individual converted to AD. As soon as the patient converted to AD, no more observations were added to this particular patient. This implies that if for example a MCI patient remained stable, we have three observations per patient. See also Table 3 for a correlation matrix of all included predictors (Table 3).

#### Model 1

The composite measure consisting of informant-rated motivation and decision regulation at baseline and the three follow-up measurement points, were the only predictors entered in the first model. Given that the model predicts the probability of MCI stability / conversion across time, the inclusion of the discrete measurement points was necessary. The results indicated that the predictors explained 53.1% (Nagelkerke *R*^*2*^) of the variance. Higher informant-rated motivational abilities significantly predicted MCI stability (OR = 0.59; *p* = 0.040) (Table 5). A comparison between the predictive strength of the two motivational components of the composite measure indicated that while motivation regulation was not found to be a meaningful predictor of MCI stability in this model (OR = 0.97; *p* = 0.133), decision regulation did significantly predict MCI stability (OR = 0.96; *p* = 0.049).

**Table 5:** Survival analyses using binary logistic regression predicting likelihood of Mild Cognitive Impairment (MCI) stability (coded as 1) (*n* = 64)

Variable	*B*	SE	*P Value*	OR	95% CI
*Model 1*					
Follow-up 1	−1.24	0.66	**< 0.001**	0.289	0.153–0.546
Follow-up 2	−1.85	0.59	**< 0.001**	0.157	0.065–0.379
Follow-up 3	−1.78	0.56	**< 0.001**	0.168	0.064–0.436
Motivational abilities ^a^	−0.53	0.36	**0.040**	0.587	0.353–0.976
*Model 2*					
Follow-up 1	−0.52	0.92	0.572	0.596	0.099–3.582
Follow-up 2	−1.85	1.05	*0.078* ^c^	0.157	0.020–1.233
Follow-up 3	−0.76	1.01	0.454	0.468	0.064–3.413
Motivational abilities ^a^	−0.65	0.33	*0.052* ^c^	0.524	0.273–1.005
Age	0.33	0.33	0.323	1.389	0.725–2.661
Sex (0 = male, 1 = female)	−0.53	0.60	0.376	0.589	0.182–1.902
Children (number)	0.67	0.30	**0.028**	1.954	1.076–3.547
*Model 3*					
Follow-up 1	−1.07	1.02	0.294	0.345	0.047–2.519
Follow-up 2	−2.34	1.13	**0.038**	0.096	0.011–0.880
Follow-up 3	−1.27	1.10	0.247	0.280	0.032–2.414
Motivational abilities ^a^	−0.74	0.35	**0.036**	0.477	0.239–0.952
Age	0.13	0.36	0.727	1.135	0.558–2.308
Sex (0 = male, 1 = female)	−0.54	0.60	0.372	0.584	0.179–1.902
Children (number)	0.73	0.32	**0.022**	2.082	1.109–3.908
Executive function ^b^	0.02	0.01	0.196	1.016	0.992–1.042
*Model 4*					
Follow-up 1	−0.11	1.89	0.955	0.899	0.022–36.773
Follow-up 2	−1.46	1.91	0.446	0.233	0.005–9.854
Follow-up 3	−0.35	1.93	0.854	0.702	0.016–30.550
Motivational abilities ^a^	−0.66	0.34	*0.051* ^c^	0.515	0.264–1.003
Age	0.31	0.34	0.374	1.358	0.692–2.667
Sex (0 = male, 1 = female)	−0.58	0.64	0.360	0.559	0.161–1.940
Children (number)	0.70	0.32	**0.031**	2.003	1.066–3.763
Education (years)	−0.03	0.11	0.804	0.973	0.781–1.212
*Model 5*					
Follow-up 1	−0.07	0.96	0.940	0.930	0.141–6.120
Follow-up 2	−1.36	1.09	0.213	0.257	0.030–2.181
Follow-up 3	−0.17	1.07	0.876	0.845	0.103–6.941
Motivational abilities ^a^	−0.63	0.33	*0.060* ^c^	0.534	0.277–1.028
Age	0.16	0.38	0.684	1.169	0.551–2.479
Sex (0 = male, 1 = female)	−0.45	0.62	0.470	0.638	0.188–2.161
Children (number)	0.73	0.33	**0.024**	2.084	1.100–3.947
MCI amnestic (0 = amnestic, 1 = non-amnestic)	−1.47	0.62	**0.018**	0.230	0.068–0.773
*Model 6*					
Follow-up 1	−0.79	1.45	0.584	0.452	0.026–7.749
Follow-up 2	−1.35	1.64	0.408	0.258	0.010–6.381
Follow-up 3	−0.14	1.58	0.931	1.146	0.052–25.359
Motivational abilities ^a^	−0.50	0.49	0.303	0.606	0.233–1.574
Age	0.78	0.52	0.135	2.181	0.784–6.064
Sex (0 = male, 1 = female)	−0.38	0.92	0.683	0.687	0.113–4.163
Children (number)	0.90	0.48	*0.060* ^c^	2.455	0.963–6.255
Cardiovascular risk factors	−3.29	1.59	**0.038**	0.037	0.002–0.837

The bold values indicate a statistically significant correlation with a *p*-value less than 0.05

*B* coefficient, *CI* confidence interval, *MCI* mild cognitive impairment, *OR* odds ratio, *p* level of significance, *SE* standard error

^a^ Composite measure including informant-rated motivational abilities at baseline (t1)

^b^ Interference score of the Stroop Color-Word Test

^c^ marginally significant

#### Model 2

In the second model, we added the control variables ‘sex’, ‘age’ and ‘number of children’. Those were all statistically different between subgroups at t1. The results indicated that the predictors explained 63.3% (Nagelkerke *R*^*2*^) of the variance. It was found that higher informant-rated motivational abilities marginally predicted MCI stability, when controlling for these control variables (OR = 0.52; *p* = 0.052). Neither ‘age’ (OR = 1.39; *p* = 0.323), nor ‘sex’ (OR = 0.59; *p* = 0.376), but ‘number of children’ (OR = 1.95; *p* = 0.028) were identified as significant predictors (Table 5). A higher number of children was thus associated with a decreased likelihood for MCI stability over 3 years. A comparison between the predictive strength of the informant-rated motivation and decision regulation showed a significant prediction of MCI stability with both components (motivation regulation: OR = 2.00; *p* = 0.042; decision regulation: OR = 1.95; *p* = 0.040).

#### Model 3

In the third model, we additionally included a measure for executive functions to test whether differences at baseline executive functions where meaningfully influencing MCI stability. The inclusion of a proxy for ‘executive function’, i.e., the interference score of the Stroop Color-Word Test, increased explained variance from 63.3 to 68.7% (*R*^*2*^). ‘Executive function’ was not meaningfully associated with MCI stability (OR = 1.02; *p* = 0.196). Higher informant-rated motivational abilities significantly predicted MCI stability in this model (OR = 0.48; *p* = 0.036). Again, ‘age’ and ‘sex’ were not meaningfully predicting MCI stability, contrary again to ‘number of children’ (Table 5). While informant-rated motivational regulation did not predict MCI stability (OR = 0.97; *p* = 0.276), ‘informant-rated decision regulation’ did significantly (OR = 0.91; *p* = 0.008).

#### Model 4

The fourth model includes, besides the main predictor of ‘informant-rated motivational abilities’ and the demographic characteristics, the predictor (years of) ‘education’. ‘Education’ was added to the predictors of *Model 2* and not to those of *Model 3*, as, given the rule of thumb of 15 observations per predictor, we are at the upper limit with 8 predictors per model and 138 available observations. ‘Education’ was included to test whether the predictive effect of ‘informant-rated motivational abilities’ is reduced when controlling for a proxy of cognitive reserve. The predictors explained a variance of 62.4% (*R*^*2*^) in this model. ‘Education’ was not found to be meaningfully related to MCI stability (OR = 0.97*; p* = 0.804). Higher informant-rated motivational abilities marginally predicted MCI stability in this model (OR = 0.52; *p* = 0.051) (Table 5). Again, as in *Model 3*: while informant-rated motivational regulation did not predict MCI stability (OR = 0.97; *p* = 0.258), informant-rated decision regulation did (OR = 0.92; *p* = 0.021).

#### Model 5

The fifth model includes, besides the main predictor of ‘informant-rated motivational abilities’ and the demographic characteristics, the predictor ‘MCI subtype’. We added ‘MCI subtype’ as predictor in addition to those included in *Model* 2 (and not to *Model 3* or *Model 4*), due to a limited number of available observations. ‘MCI subtype’ was added as predictor as the clinical phenotype ‘MCI amnestic’ is commonly related to a higher conversion rate. Due to an unequal distribution of participants to the four MCI phenotypes, we combined the two amnestic and the two non-amnestic subtypes each into one group, resulting in a dichotomous MCI subtype variable (amnestic / non-amnestic). The inclusion of ‘MCI subtype’ increased explained variance from 63.3 to 66.1% (*R*^*2*^). The clinical phenotype ‘MCI amnestic’ was found to be associated with a decreased likelihood for MCI stability over 3 years (OR = 0.23; *p* = 0.018). The informant-rated motivational abilities marginally predicted MCI stability in this model (OR = 0.53; *p* = 0.06) (Table 5). While informant-rated motivational regulation did not predict MCI stability (OR = 0.97; *p* = 0.296), informant-rated decision regulation did (OR = 0.93; *p* = 0.021).

#### Model 6

We additionally calculated *Model 2* by adding a proxy for ‘cardiovascular risk‘. The predictors in this model explained 80% of the variance (*R*^*2*^). The variable ‘cardiovascular risk‘was associated with a decreased likelihood for MCI stability over 3 years (OR = 0.04; *p* = 0.038). The informant-rated motivational abilities did not predict MCI stability in this model (OR = 0.61; *p* = 0.303) (Table 5), neither did the informant-rated motivational regulation (OR = 0.96; *p* = 0.384) nor the informant-rated decision regulation (OR = 0.96; *p* = 0.249).

### Multilevel modeling

At first, we calculated whether the proportion in the variance of cognitive abilities, as measured with the MMSE, indicated the need for analyses with multilevel modeling. The intraclass correlation (ICC) was 43.90%, indicating that around 44% of the total MMSE variance resides between patients and around 56% within patient’s repeated measures. The relatively large ICC indicates that data can be analyzed by multilevel modeling [53].

We found a significant between individual variance (Wald *Z* = 4.22, *p* < 0.001), indicating significant variation in the intercepts of MMSE (= random intercept; AIC = 1177). We also found a significant variation in the slope of MMSE between participants (= random slope; AIC = 1067; Wald *Z* = 4.33; *p* < 0.001). As of that, further analyses were conducted using the random intercept, random slope model. Additionally, we calculated the model with quadratic time to test for curvilinearity in the trajectory of MMSE. The effect was not significant (*p* = 0.113). Consequently, we calculated our models with linear time only.

In order to provide comparative analyses to the survival analyses models using logistic regression above, we calculated the multilevel models without interaction effects. Nonetheless, interaction effects of time with all predictors were calculated for all models (data not shown). The addition of interaction effects did not result in increasing the model fit as indicated by the model fit index AIC.

#### Model 1

In the first model, we were interested in the main effect of ‘informant-rated motivational abilities’ on the trajectory of cognitive abilities over a three-year observation period. As of that, we included only time and ‘informant-rated motivational abilities’ into the first model. We found a significant negative main effect of ‘time’ (*t* = − 3.08; *p* = 0.002) and significant positive effect of ‘informant-rated motivational abilities’ (*t* = 2.33; *p* = 0.023) on the trajectory of cognitive abilities.

#### Model 2

In a second model, we added the demographic characteristics ‘age’ and ‘sex’. Unlike the survival analyses, we did not add ‘number of children’ in these models, as current literature is not suggestive of a protective influence of having fewer children on the course of cognitive abilities. Again, we found a significant negative main effect of ‘time’ (*t* = − 3.08; *p* = 0.002). In addition, we found a marginal positive effect of ‘informant-rated motivational abilities’ (*t* = 1.95; *p* = 0.056). The effect of the demographic characteristics ‘age’ (*t* = − 1.21; *p* = 0.231) and ‘sex’ (*t* = 1.54; *p* = 0.129) were not significant.

#### Model 3

In the third model, we included a proxy for ‘executive function’. We found a significant negative main effect of ‘time’ (*t* = − 3.28; *p* = 0.002), a significant positive effect of ‘informant-rated motivational abilities’ (*t* = 2.32; *p* = 0.023), and a significant negative effect of the interference score of the Stroop Color-Word test (*t* = − 3.03; *p* = 0.004), which can be translated into a significant positive effect of ‘executive functions‘. ‘Age’ (*t* = -0.59; *p* = 0.558) and ‘sex’ (*t* = 1.64; *p* = 0.106), were again not significant in this model.

#### Model 4

In the fourth model, we included a proxy for cognitive reserve, i.e., years of ‘education’. We found a significant negative main effect of ‘time’ (*t* = − 3.28; *p* = 0.002), a significant positive effect of ‘informant-rated motivational abilities’ (*t* = 2.36; *p* = 0.022), and a significant negative effect of the interference score of the Stroop Color-Word test (*t* = − 2.80; *p* = 0.007), which, again, can be translated into a significant positive effect of ‘executive functions‘. ‘Age’ (*t* = -0.59; *p* = 0.560) and ‘education’ (*t* = 0.40; *p* = 0.689) were not significant, and ‘sex’ only marginally (*t* = 1.68; *p* = 0.098).

#### Model 5

In the final model, we additionally included ‘MCI subtype’. We found a significant negative effect of ‘time’, a significant positive effect of ‘informant-rated motivational abilities’, a significant negative effect of the interference score of the Stroop Color-Word test, i.e., significant positive effect of ‘executive functions‘(see Table 6). The R^2^ estimate for this final and best fitting model is 41.03.

**Table 6:** Growth model parameter estimates of predictors for trajectories in cognitive abilities, as measured with the Mini Mental State Examination (MMSE) (*n* = 64)

Variable	MMSE					
	Fixed effects				Random effects	
	Estimate (s.e.)	*df*	*t*	*p*	Variance	Co-Variance
Intercept	26.11 (0.71)	63.73	26.80	**0.001**	1.26	−0.45 ^a^
Time (years)	− 0.82 (0.25)	54.74	−3.29	**0.002**	2.96	−0.45 ^a^
Motivational abilities ^b^	0.56 (0.25)	60.90	2.23	**0.030**		
Age	−0.11 (0.23)	64.39	−0.49	0.623		
Sex (0 = male, 1 = female)	0.76 (0.48)	61.83	1.58	0.120		
Executive function ^c^	− 0.62 (0.22)	60.85	−2.78	**0.007**		
Education (years)	0.08 (0.24)	62.15	0.32	0.751		
MCI subtype (0 = amnestic, 1 = non-amnestic)	0.16 (0.47)	62.92	0.35	0.731		
Residual					2.57	
*AIC = 1047.86*						

The bold values indicate a statistically significant correlation with a *p*-value less than 0.05

*AIC* Akaike information criterion, *df* degrees of freedom, *MMSE* Mini-Mental State Examination, *p* probability, *s.e.* standard error, *t* independent t-test

^a^ Covariance of the Intercept and Time random effects

^b^ Composite measure including informant-rated motivational abilities at baseline (t1)

^c^ Interference score of the Stroop Color-Word Test

#### Additional analyses

We also compared the predictive strength of informant-rated motivation and decision regulation in *Model 5*. We found that while ‘motivation regulation’ was not a meaningful predictor (*t* = 0.92; *p* = 0.361), ‘decision regulation’ had a significant positive effect on cognitive abilities in this model (*t* = 3.03; *p* = 0.004). Also, we added the predictor ‘depression’ to *Model 5* in order to control for influences of depressive mental states. ‘Depression’ predicted cognitive abilities significantly (*t* = 2.10; *p* = 0.04). Motivational abilities remained a significant predictor in this model (*t* = 3.23; *p* = 0.002). Finally, we added a proxy for health (‘cardiovascular risk’) to *Model 5* in order to control for the effect of health. While the measure for ‘cardiovascular risk’ did not significantly predict cognitive abilities (*t* = 1.30; *p* = 0.201), the inclusion of this somatic risk factor decreased the predictive ability of ‘informant-rated motivational abilities’ to a non-significant effect (*t* = 1.15; *p* = 0.259).

**Fig. 1:**
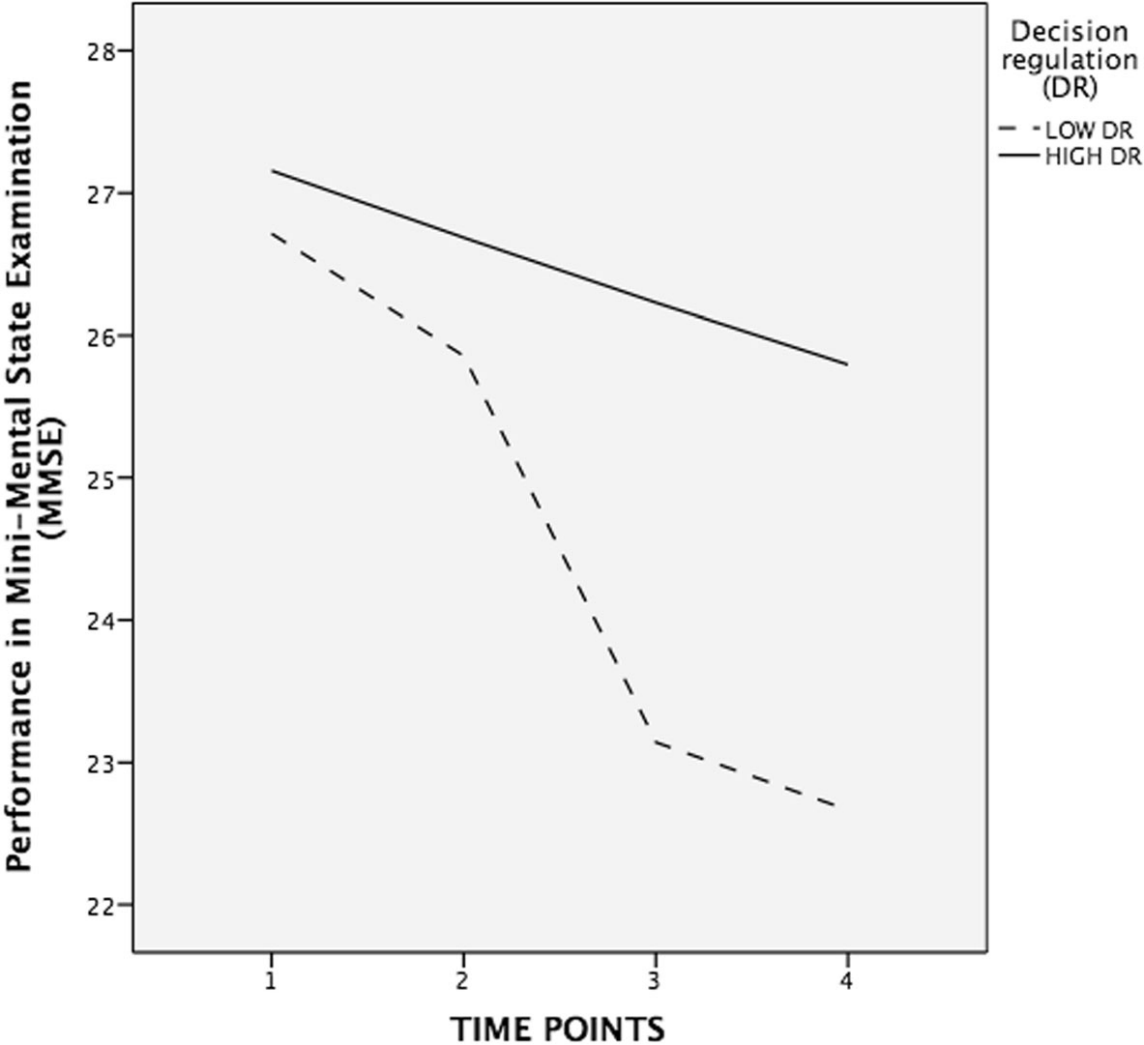
Trajectories in performances in Mini-Mental State Examination (MMSE) over the four measurement time points for individuals with high and low informant-rated decision regulation, computed with median split

Figure 1 illustrates the differential trajectories of individuals with high and low decision regulation, as calculated with median split, over the four measurement time points with regard to their performances in the MMSE (Fig. 1).

## Discussion

Here we aimed to examine the hypothesized protective influence of motivational abilities on the course of MCI. In the context of a longitudinal, prospective study, we compared the predictive effect of informant-rated motivational abilities measured at baseline on the likelihood of MCI stability over a time period of 3 years. Our main finding was that informant-rated motivational abilities were meaningful predictors of MCI stability, even when controlling for demographic characteristics, executive functions at baseline or MCI subtype (i.e., amnestic vs. non-amnestic). The analyses conducted with logistic regression were partly supported by analyses using multilevel modeling. A comparison of the predictive strength of motivation versus decision regulation suggests that the potentially protective effect of motivational abilities may mainly be driven by the motivational ability ‘decision regulation’. However, the control for the influence of cardiovascular risk at baseline resulted in the disappearance of a meaningful effect of motivational abilities. Given the relatively small sample size and the related low statistical power, in addition to the occasionally only marginally significant findings, our results may be considered as preliminary.

In our sample, we observed a conversion rate from MCI to AD of 32.8%. Given the relative short observation window of 3 years, this rate can be considered as comparatively high [4]. Previous studies have indicated that individuals who seek professional help due to their memory impairments [57], as well as individuals who complain about their memory loss [58], are at a higher risk for conversion. Given that we were recruiting our participants in specialized clinics and institutions, as well as by advertisements, we might have recruited individuals at a higher risk for conversion.

Our preliminary finding of a meaningful prediction of MCI stability by informant-rated motivational abilities corroborates previous cross-sectional [30] and prospective findings [31]. Given that in both previous studies, an indirect measure of motivational abilities (i.e., an estimate for motivational abilities that is based on the main occupation the individual had in midlife, by applying the O*NET, see description in [30, 31, 41, 59]) was used to predict MCI courses, we could extend those finding by showing that informant-rated motivational abilities can be used to predict MCI courses. We re-calculated all analyses with self-rated motivational abilities (data not shown). We found that self-rated motivational abilities are not effective in predicting the course of MCI stability. This corresponds to findings by Snow and colleagues, who concluded that self-reports of individuals with cognitive decline may be less accurate due to a diminished awareness [40].

On the basis of our analyses, it appears as if the motivational ability ‘decision regulation’ may be more closely related to MCI stability and cognitive abilities as compared to ‘motivation regulation’. Decision regulation may be linked to the actual behavioral decision-making process. A recent longitudinal study with non-demented older adults (mean age of 83.5 years) found that poor decision-making reflected deteriorating cognitive abilities [60], rather than predicting cognitive decline. The authors concluded that their results “..suggest that even very subtle age-related changes in cognition have detrimental effects on judgment.” [60]. As a consequence, it may be that in the current study, differences in the informant ratings of decision regulation may reflect subtle, indirect judgments of cognitive decline, more so than differences in motivation regulation.

The predictive ability of motivational abilities disappeared when controlling for the influence of cardiovascular risk factors. It can be assumed that cardiovascular risk might act as a mediator between motivational and cognitive abilities by translating motivational abilities into beneficial health outcomes. For instance, higher motivated individuals may engage more in activities that are promoting cardiovascular health, such as the increased engagement in physical activities, which are known to exert a beneficial effect on cognitive health in older age (e.g. [61]). How can it be explained that motivational abilities predict a more benign course of MCI? Analogous to the brain or cognitive reserve concepts [8–10, 28], we propose the motivational reserve concept, based upon the assumption that the increased use of certain brain areas promotes synapse formation and neuronal growth, which lead to higher efficiency within the brain, that in turn allows the compensation of impaired neuronal networks. By an increased activation of brain areas directly related to motivational abilities, such as the prefrontal cortex, the nucleus accumbens and the amygdala [62, 63], or indirectly related by the influence of motivation on stress, emotional health or cognition [30, 31], those areas become more efficient and are thus able to compensate for affected networks through brain plasticity. Our preliminary findings support the predictions drawn from the motivational reserve model by showing that higher motivational abilities at an early stage of MCI may exert a protective influence on the course of MCI. Future studies are needed to investigate the underlying mechanims (e.g. potential mediation by health behavior tendencies) in the translation of motivational abilities into better health outcomes in later life.

An unexpected finding was the meaningful, *negative* association between motivational abilities and education, as it was expected that a higher level of motivational abilities would be related to higher educational achievements (i.e., more years of education). When controlling for the impact of gender, this association disappears. The control for the influence of gender might in fact be relevant with regard to this relation: on one hand, in our sample, female gender was related to higher motivational abilities. On the other hand, Swiss women of the generation of our sample (cohorts 1930–1960) did not gain the same access to higher education as their male counterparts. This is also reflected in the significant difference in years of education between male and female participants. As of that, it may be that a (social) gender bias within our older sample might have obscured the expected meaningful association between motivational abilities and (years of) education.

A further unexpected finding of our study was the result of a lower risk for MCI conversion in individuals having fewer children. Given the fact that we included this variable as a control variable due to a significant difference between the two MCI subgroups, this inclusion was not led by theory. To the best of our knowledge, this finding is unprecedented. A higher number of children is an indirect indicator of lower parental intelligence [64] and socio economic status (SES). Thus, we speculate that those individuals having more children may have less protection against cognitive decline, as higher intelligence and SES have a preventive influence on pathological cognitive decline [9, 10, 65]. However, our data did not reveal a meaningful relation between number of children and intelligence or years of education (as a proxy for SES), neither for men nor women (all correlations are non-significant, data not shown). Another, though highly speculative explanation, may be found in the life history theory (LHT), that states that increased reproductive effort (i.e., a higher number of offspring), might come at the expense of tissue maintenance and thus accelerate aging [66]. For instance, in a study including older women (mean age of 75 years), it was found that a higher number of children was associated with shorter telomere length [67], a proxy for cellular aging that has previously been linked to dementia risk [68]. However, a recent, prospective study did not find a meaningful association between the number of children and maternal telomere length. Rather, mothers with fewer children had even shorter telomere lengths [69]. Again, this explanation is highly speculative and more research is needed to understand the unexpected finding in our study that a greater number of children was associated with higher risk for MCI conversion.

Our findings must be interpreted in the light of the following limitations. A limiting factor of our study is the use of current motivational abilities that have been rated by informants of the participants. The assessment of current motivational abilities is flawed by the fact that it can be argued that lower current motivational abilities may be an early sign of an underlying neuropathological process rather than a premorbid risk factor. In fact, pathological cognitive decline seems to impact motivational abilities. For instance, in an own study, we were able to show that individuals with mild AD displayed higher rates in the motivational construct ‘delay discounting’ over an observation period of 2 years which indicates decreasing self-control with progressive cognitive decline [70]. Brain areas related to motivational abilities, such as the prefrontal cortex, the nucleus accumbens and the amygdala [62, 63] can all be affected by AD neuropathological processes. It is as such a possibility that lower motivational abilities in our study do reflect some sort of cognitive decline. However, this may not completely undermine our hypothesis of a protective effect of higher motivational abilities on the course of MCI, as higher levels of motivational abilities may still act as a buffer against cognitive decline, even when impairment has already begun. Nonetheless, we may not completely exclude the argument that lower current motivational abilities reflect early AD disease processes.

The optimal predictor with regard to the research question of interest may have been the direct assessment of motivational abilities during midlife, i.e., decades before the first clinical signs of cognitive impairment appeared. Given that this was not feasible in the current study, other possibilities (besides the indirect assessment through estimates of motivational abilities on the basis of participants’ occupational history, see [30]), were either the retrospective assessment, which, however, is flawed by recall bias, or the use of the rating by informants, which, as we were able to show in a previous study, can be biased by the caregiving burden [71].

A further essential limiting factor is the small sample size of our study, particularly with regard to the regression analyses. Those results have to be interpreted with caution. Due to the small sample size and the related low statistical power [72], it is both likely that we did not find significant effects of factors that were previously identified as risk (MCI subtype) or protective factors (i.e., education). It is also likely that due to the small sample size our significant finding of a protective effect of motivational abilities on the course of MCI may not mirror a true effect [72], i.e., could have been an overestimation. In combination, i.e., due to the fact that our independent variable might have been affected by ongoing underlying disease processes, in combination with the small sample size, the external validity of our findings is limited, and interpretations of our results should be done with caution.

What is more, we did not assess the time point of when the diagnoses of the patients were made for the first time, which could have been used as a standardized proxy of the onset of the clinical symptoms of MCI. While most of our participants were diagnosed in the months before recruitment for the study, we have no information with regard to how long MCI symptomatology persisted before participants presented themselves to a clinician and were then diagnosed with MCI. It could therefore be that patients with stable courses were those with the most recent onset of MCI. But given the fact that the pre-clinical biological changes related to AD might take years to decades before the clinical phase of AD [73], any ‘starting point’ could only ever be vaguely defined. Nonetheless, future studies should include duration of the clinical manifestation of the symptomology as a proxy. Future studies should also include the assessment of biomarkers, to increase the level of certainty of the AD diagnosis [2]. Also, the assessment of A*B* protein would have been useful as it has previously been proposed that A*B* negative MCI cases are unlikely to progress to AD [1]. Furthermore, our measure for ‘health’, i.e., a proxy for cardiovascular health, is limited. Future studies should include a broader indicator for physical health. Additionally, it is to be assumed that the participants (and their informants) show self-selection bias. Taking part in a relative extensive longitudinal study while being diagnosed with MCI may itself be a sign for higher motivational abilities. Therefore, it may be that our results are biased towards higher motivated, research interested participants, which might have affected our study on various levels (e.g. recruitment, data collection) and might thus reduce validity of our results. Also, the assessments of the diagnoses at follow-ups were carried out by a group of clinicians that might involve the same or other persons that carried out the diagnoses at baseline. However, this group of clinicians was *not* involved in the assessment of affective and motivational variables. It was strived for having the group of clinicians blinded for the previous MCI and AD diagnoses, however, this could not have been guaranteed for all participants. Finally, the observation period of 3 years is relatively short. It is thus of importance to replicate our findings in a study with a larger sample size that is followed up for a longer period of time.

## Conclusions

Bearing in mind the preliminary nature of our results, this study provides first evidence for a protective effect of motivational abilities on the course of MCI. Given the modifiable nature of motivational abilities, future studies should investigate how those skills may best be trained in coaching courses, how they might be integrated into existing psychotherapeutic manuals for both older and younger individuals and whether such interventions are effective in increasing cognitive health in older age [31].

## Abbreviations

AB: amyloid-beta
AD: Alzheimer’s disease
CDR: Clinical Dementia Rating Scale
CERAD-NP: Consortium to Establish a Registry for Alzheimer’s Disease – Neuropsychological Assessment
CR: Cognitive reserve
DSM-IV-TR: Diagnostic and Statistical Manual of Mental Disorders
GDS: Geriatric Depression Scale
LHT: Life history theory
MA: Motivational abilities
MCAR: Little’s missing completely at random
MCI: Mild cognitive impairment
MMSE: Mini Mental State Examination
MoReA: Motivational Reserve as a Protective Factor in Mild Alzheimer’s Dementia and Mild Cognitive Impairment
MR: Motivational reserve
N: Number
NINCDS-ADRDA: National Institute of Neurological and Communicative Disorders and Stroke-Alzheimer’s Disease and Related Disorders Association
O*Net: Occupational Information Network
SES: Socio economic status
VCQ: Volitional Components Questionnaire
